# Children of extremist parents: Insights from a specialized clinical team

**DOI:** 10.1177/13591045231192340

**Published:** 2023-08-04

**Authors:** Cécile Rousseau, Diana Miconi, Janique Johnson-Lafleur, Christian Desmarais, Ghayda Hassan

**Affiliations:** 1 Department of Psychiatry, 5620 McGill University, Montreal, QC, Canada; 2 Educational Psychology and Andragogy, 5622 University of Montreal, QC, Canada; 3 Sherpa University Institute, 12367 McGill University Faculty of Medicine, Montreal, QC, Canada; 4 Department of Psychology, 98643 Universite du Quebec a Montreal Faculte des Sciences, QC, Canada

**Keywords:** Violent extremism, parents, clinical services, stereotypes, child protection

## Abstract

**Background:**

Data on children who grow up with parents adhering to violent extremism is scant. This makes it extremely delicate to inform policies and clinical services to protect such children from potential physical and psychological harm.

**Objective:**

This paper explores the predicament of children whose caretakers were referred to a specialized clinical team in Montreal (Canada) because of concerns about risks or actual involvement in violent extremism processes.

**Methods:**

This paper uses a mixed methods concurrent triangulation design. Quantitative data was obtained through a file review (2016–2020). Qualitative data was collected through semi-structured interviews and a focus group with the team practitioners.

**Results:**

Clinicians reported the presence of stereotypes in the health and social services network frequently representing religious extremist parents as potentially dangerous or having inappropriate parenting skills while minimizing the perception of risk for parents adhering to political extremism. Children displayed high levels of psychological distress, mainly related to family separation, parental psychopathology, and conflicts of loyalty stemming from familial or social alienation.

**Conclusions:**

Training practitioners to be aware of their own personal and institutional bias may help them to understand the predicament of extremist parents’ children and implement systemic, trauma and attachment informed interventions.

In 2021, the United Nations Special Rapporteur stated that “the interaction between family regulation and counter-terrorism is accelerating with profound implications for both” (Human Rights Council, 2021). Although the legal regulation of family life in the name of National Security emerges as a timely area of concern, balancing political and clinical implications to protect the best interests of children and protect them from physical and psychological harm is an extremely difficult and delicate task given the paucity of data available on children who grow up with parents adhering to a form of violent extremism. In an effort to begin filling this gap in knowledge, this paper explores the predicament of children whose caretakers were referred to a specialized clinical team in Montreal (Canada) because of concerns about risks or actual involvement in violent extremism processes.

Despite its relevance, the impact of parental ideology and engagement with violence on children is still a largely under-researched domain. After the Second World War, studies on children of Nazis (Bar-On, 1989) suggested that parent-child transmission of Nazi ideologies was often buffered by the normalization of the past and by silence around parental involvement. In a rare empirical study on former extremist parental influences on the radicalization of their children (Sikkens et al., 2017), parents and children overall did not recognize a direct influence of parents on radicalization processes in children. However, these results may reflect the same kind of minimization and denial as after World War II and the authors did not look for possible developmental or mental health consequences in the children of extremist parents. Given the complexity of those processes, it is possible to envision that these other consequences may vary as a function of collective meaning attributed to parental engagement, parental psychopathology, family dynamics (including domestic violence) and attachment patterns. This is what a recent research on offspring of child-soldiers in Africa (Burundi) suggests. This study showed that trauma was transmitted from the parents to their children through the cultural patterns of parenting and education called “Indero,” and through community effects and parental psychopathology (Song et al., 2014). Beyond the parents and families themselves, extremist organizations may encourage their members to have children and to raise them according to the group ideology in order to increase their resources and influence (Koehler, 2020). The ideology of the group may be inculcated through harsh discipline, hate values and different forms of indoctrination in white supremacist movements (Simi, 2010) as well as in Jihadist terror organizations (Horgan et al., 2017; Riany et al., 2019). Empirical research has yet to investigate these possibilities, but the difficulties of conducting research with hard to reach and distrustful subjects have for the moment limited available knowledge.

Recently, interest in children of extremist parents has been awakened by the mass recruitment of young adults by DAESH, and the publicity of the Caliphate about the education they provide to children (Spyra, 2020). A lot of political and media attention has been given to foreign fighters’ children, in the camps and/or as returnees, depicting them either as traumatized (Weine et al., 2020) and/or as potential aggressors (Pašagić, 2019). This has led many nations to refuse the return of such children, in spite of the fact that such a position was contravening numerous international conventions, and in particular the Convention on the Right of the Child (CRC, 1989; MacVicar, 2020). In a unique study conducted in France on 47 children, all separated from their parents, Mapelli et al. (2019) report that children (66% younger than 6 years) presented post-traumatic stress disorder (PTSD), attachment problems, anxiety, depression, sleeping and eating difficulties. Clinicians were challenged by the institutional mandate they received from the government which interfered with standard care and with the decision-making process in the best interest of the child (Guerin Franchitto, 2021). They also highlighted the difficulty of providing care to such children without a proper anamnesis (Klein et al., 2020). Overall, although the foreign fighter children represent a relatively small number of children, their politically sensitive predicament has led to the production of several guidelines for practitioners (Weine et al., 2020). However, given the paucity of data in the field, such guidelines are based on empirical evidence in related fields (for example childhood trauma and child soldiers) and have not been evaluated yet in terms of their efficacy for this specific clientele. In light of extensive research associating the implementation of Countering Violent Extremism (CVE) policies (Weine et al., 2020) with harmful profiling of Muslim families (parents and children), the potential danger of these policies for families has been recently denounced by international organizations (Human Rights Council, 2021). Even less is known about new “homegrown” forms of violent radicalization which are on the rise in Western societies, including groups associated with the far-right, such as the white-supremacists, the Neo-Nazis and some libertarian and anti-system groups advocating for the use of violence.

To the best of our knowledge so far, there is no research or clinical literature describing the situation of children of parents adhering to the new forms of violent extremism associated with the majority in North America and Europe, in spite of the fact that they are now recognized as a major social threat in the US (Allam and Nakashima, 2021) and in Canada.

In summary, very little is known yet about the children of extremist parents and the present polarization in the field may introduce a bias which could exaggerate the risks in targeted minorities and minimize or ignore it in groups which identify with the majority, mirroring the blind-spots and harmful consequences of initial CVE policies which resulted in the stigmatization of minority groups (in particular Muslim communities).

Analysing the clinicians’ perspectives and chart-review data about children of extremist parents collected by a specialized team in Montreal, Quebec, this mixed-method paper aims to describe the consequences of extremist parents’ involvement for their children and youth and to identify some main clinical and legal challenges which require future attention.

## Methods

### Setting: The Polarization team

In 2016, Quebec implemented an innovative intervention model for extremist individuals comprising of: (1) a multiple entry point system; (2) coordination of local specialized services with expertise in cultural psychiatry and violence risk assessment and management; and (3) a strong coordination with proximity services, including a mentoring program focusing on social integration and life-skills. This clinical model is tailored to be flexible and adapt to populations with diverse characteristics and to local resources. The Montreal clinical team, called Polarization provides services to extremist individuals, their significant others, and to some victims of hate crimes. Polarization, in partnership with external researchers, has launched an evaluative mixed method research to document diverse aspects of its clinical work.

### Mixed method

This paper uses a mixed methods concurrent triangulation design (Creswell and Plano Clark, 2007) to complement available quantitative data on children of extremist parents with qualitative data collected from clinicians working within a specialized team.

• **Quantitative:** retrospective file review material (total *n* = 160). **Inclusion criteria**: being a minor aged 0-17 yo and having at least one extremist parent assessed and/or followed by the Polarization team. Indirect consultations demanded to the team by a child caretaker other than the parents were also included; **exclusion criteria**: extremist\radicalized minor (less than 18 years old) in a family without identified extremist parents.
• **Available variables**: age, gender, type of extremism, parental diagnosis, children diagnosis, type of clinical engagement (assessment and follow-up of children, assessment and follow-up offered to parents, indirect consultation about children\families to other practitioners). 23 files corresponded to the inclusion criteria. Because of the small sample and the highly sensitive confidentiality issues data is presented as aggregated.
• **Analysis**: Descriptive information for the sample was summarized using means and standard deviations for continuous variables and counts and proportions for categorical variables, in the overall sample and by age group (see Table 1). Pearson’s chi-square tests of independence were performed in R (R Development Core Team, 2017) to examine the association between age group (0–5; 6–11; 12–17), type of extremism (religious vs non-religious) and child’s type of clinical engagement (assessed vs non-assessed by the team). *Cramer’s V* was used as a measure of effect-size.
• **Qualitative:** Semi-structured individual interviews were conducted with clinical service providers and clinical supervisors (*n* = 5) providing social polarization services in the Montreal team. A focus group (*n* = 7) was also conducted by videoconference with the team clinicians. For both individual and group interviews, the interview guide focused on clinicians’ perceived barriers and facilitators to reaching out, engaging, and providing clinical services to children of extremist individuals, and their experience and perspectives on working with families with extremist parents. The rationale of combining individual interviews and a group interview was to enhance the richness of the data by accessing both collective and personal ways of addressing and elaborating on the issues under study. The interviews were audio-taped and transcribed verbatim. A thematic analysis (Braun and Clarke, 2019) was performed on the collected material with the assistance of NVivo 12. Ethical approval was obtained from the Research and Ethics Board of the CIUSSS-CODIM. All material was cautiously examined and censored to avoid any confidentiality breech.
• **Mixed analysis**: The results of the quantitative and qualitative analyses were compared in terms of convergence, complementarity, and divergence. A mixed methods matrix technique (O’Cathain et al., 2010) was used to integrate qualitative and quantitative data and to allow for a more comprehensive understanding of the study topic, including the dynamics of collaboration or non-collaboration between the various family members and professional partners.

**Table 1.** Characteristics of Participants in the Total Sample and by Age Group (*N* = 23).

	Preschool (0–5) (*N* = 8)	Primary school (6–11) (*N* = 11)	Secondary school (12–18) (*N* = 4)	Total (*N* = 23)
Gender				
Girls	6 (75.0%)	5 (45.5%)	2 (50.0%)	13 (56.5%)
Boys	2 (25.0%)	6 (54.5%)	2 (50.0%)	10 (43.5%)
Age				
Mean (SD)	4.13 (.641)	7.73 (1.68)	14.3 (2.87)	7.61 (3.87)
Median [min, max]	4.00 [3.00, 5.00]	8.00 [6.00, 10.0]	13.5 [12.0, 18.0]	6.00 [3.00, 18.0]
Type of extremism				
Conspirationist/anti-system	1 (12.5%)	1 (9.1%)	0 (0%)	2 (8.6%)
Extreme right	0 (0%)	3 (27.3%)	1 (25.0%)	4 (17.4%)
Masculinist/gender	1 (12.5%)	0 (0%)	0 (0%)	1 (4.3%)
Religious	6 (75.0%)	5 (45.5%)	3 (75.0%)	14 (60.9%)
Supremacist/masculinist	0 (0%)	2 (18.2%)	0 (0%)	2 (8.7%)
Type of extremism (R)				
Religious extremism	6 (75.0%)	5 (45.5%)	3 (75.0%)	14 (60.9%)
Other non-religious extremism	2 (25.0%)	6 (54.5%)	1 (25.0%)	9 (39.1%)
Was the child assessed?				
No	6 (75.0%)	6 (54.5%)	1 (25.0%)	13 (56.5%)
Yes	2 (25.0%)	5 (45.5%)	3 (75.0%)	10 (43.5%)
Was the parent assessed?				
No	4 (50.0%)	1 (9.1%)	0 (0%)	5 (21.7%)
Yes	4 (50.0%)	10 (90.9%)	4 (100%)	18 (78.3%)
Was indirect consultation provided?				
No	4 (50.0%)	10 (90.9%)	4 (100%)	18 (78.3%)
Yes	4 (50.0%)	1 (9.1%)	0 (0%)	5 (21.7%)
Was the parent diagnosed?				
No	4 (50.0%)	1 (9.1%)	1 (25.0%)	6 (26.1%)
Yes	4 (50.0%)	10 (90.9%)	3 (75.0%)	17 (73.9%)
Was the child diagnosed?				
No	6 (75.0%)	6 (54.5%)	1 (25.0%)	13 (56.5%)
Yes	2 (25.0%)	5 (45.5%)	3 (75.0%)	10 (43.5%)
Parent’s diagnosis				
Bipolar disorder	1 (12.5%)	0 (0%)	0 (0%)	1 (4.3%)
Borderline personality disorder	1 (12.5%)	3 (27.3%)	0 (0%)	4 (17.4%)
Complex PTSD	1 (12.5%)	1 (9.1%)	0 (0%)	2 (8.7%)
Personality disorder	1 (12.5%)	3 (27.3%)	1 (25.0%)	5 (21.7%)
Psychosis/Depression	0 (0%)	1 (9.1%)	2 (50.0%)	3 (13.0%)
Psychosis/PTSD	0 (0%)	2 (18.2%)	0 (0%)	2 (8.7%)
Missing	4 (50.0%)	1 (9.1%)	1 (25.0%)	6 (26.1%)
Child’s diagnosis				
Adjustment disorder	0 (0%)	0 (0%)	1 (25.0%)	1 (4.3%)
Anxiety	0 (0%)	0 (0%)	1 (25.0%)	1 (4.3%)
Attatchment disorder	0 (0%)	2 (18.2%)	0 (0%)	2 (8.7%)
Attachment	1 (12.5%)	0 (0%)	0 (0%)	1 (4.3%)
Attachment disorder/ADHD	0 (0%)	1 (9.1%)	0 (0%)	1 (4.3%)
Conduct disorder	0 (0%)	1 (9.1%)	0 (0%)	1 (4.3%)
DI, anxiety	0 (0%)	0 (0%)	1 (25.0%)	1 (4.3%)
None	0 (0%)	1 (9.1%)	0 (0%)	1 (4.3%)
PTSD	1 (12.5%)	0 (0%)	0 (0%)	1 (4.3%)
Missing	6 (75.0%)	6 (54.5%)	1 (25.0%)	13 (56.5%)

The Research Ethics Boards of The Centre intégré universitaire en santé et services sociaux de l’Ouest-de-l'Île de Montréal approved the file review study and the practitioner’s perspective study. Participation of practitioners was informed, voluntary, and anonymous. All data was carefully reviewed to ensure confidentiality.

## Results

### Quantitative results

In total, 23 out of 160 files met the inclusion criteria (14%). Children mean age was 7.5 years (*SD* = 3.87, range 3–17 years old) (Table 1). In terms of gender, 43% of the children were boys and 57% girls.

With regards to parents’ extremism, 14 were referred for religious extremism and 9 for other forms of extremism ranging from extreme right, white supremacist, masculinist and conspirationist. Among these 23 children, ten became clinically engaged because of a legal child protection prescription, all of which had parents presenting religious extremism. In four cases, support was provided to the extended family of foreign fighters’ children, without direct contact with the parents or the children themselves.

Parental diagnosis was available for 17 parents, who sometimes received multiple diagnosis. Most common diagnosis among parents were personality disorders (BPD and narcissistic), psychosis (delusional or affective), Complex Post Traumatic Stress Disorder (CPTSD) and Post Traumatic Stress disorder (PTSD).

Diagnosis was available for 10 children, with a predominance of stress related disorders (anxiety\adjustment disorder\PTSD) and attachment disorders.

Results from chi-squared tests showed that the proportion of children who were assessed by the team was larger among religious extremist parents (*n* = 10) than among parents who adhered to non-religious forms of extremism (*n* = 0) (χ^2^ (df) = 8.65 (1); *p* = .003, *Cramer’s V* = .61). No significant age differences emerged across types of extremism (χ^2^ (df) = 2.10 (2); *p* = .349, *Cramer’s V* = .30) or type of children’s clinical engagement (χ^2^ (df) = 2.75 (2); *p* = .253, *Cramer’s V* = .35).

### Qualitative results: Practitioners’ perspective

#### Engagement and therapeutic alliance

Clinicians reported that most parents were initially distrustful of health services and accepted that their children be assessed and followed only because they had no choice (if youth protection was involved), or in the hope to appear favorably in court procedures or in front of security agencies, as they were convinced that the team would report to these, in spite of having been told the contrary. According to the clinicians, the parents would often oscillate between contrasting positions. Sometimes they would adopt a submissive stance, trying to conform or to seduce the clinicians by portraying themselves as helpless victims. In other moments, they expressed anger, voiced that they felt they were being unfairly treated and opposed any form of help for their children (speech therapy assessment, neuro-psychological testing, or homework support). Other parents would mainly avoid clinical encounters by forgetting and repeatedly cancelling appointments. Finally, two parents would invest positively the therapeutic setting and use it to comfort a power position toward the other parent which they would accuse of being more radicalized, and in one case to psycho-pathologize the distress of their child, the diagnosis playing a protective role to relieve the guilt related to a very insecure attachment.

When parents were distrustful, clinicians felt that children experienced ambivalence toward assessment or therapy, being caught in a conflict of loyalty. In that situation most school-aged children and adolescents would carefully avoid sensitive topics, and in particular talking about home or about their parents. Sometimes they would acknowledge how difficult this was, like Mary sharing “My parents are quite something…But I love them”. Younger children (age 4–7 years old) would disclose a lot of their personal predicament through symbolic play, sometimes revealing information and being scolded by their siblings for doing so, in the case of joint therapy sessions. A young girl displayed elective mutism for months before beginning to engage into therapy. Finally, according to two clinicians, symbolic play allowed the children to express indirectly attachment to the therapist without feeling that they were betraying their parents.

Practitioners reported that clinical work with caretakers of foreign-fighters’ children (pre-schoolers) was rewarding when they had the impression they could help to de-stigmatize the situation and support the re-establishment of a secure and caring environment for the child. In some cases, however the pain and despair of the caretakers, unable to speed up reunification procedures and to protect the child, was a source of helplessness in the clinicians.

Clinicians also commented on their multifaceted role and the range of strategies they employed to engage the parents and the children. Initially, all of them agreed on the importance of being clear around the robust legal firewalls which exist in Canada between health professionals and security agencies, insisting on the fact that no information would be forwarded to the security agencies unless there was a direct and immediate threat for life. They reported knowing that this was not enough to build trust, which often required months or years to establish. Home visits were always proposed. Some parents were pleased to receive the clinicians as a way of establishing a more balanced relation through the rituals of hospitality. Others felt threatened by what they perceived as a further intrusion in their lives. Last, some parents accepted the home visit, but made sure the children would not be seen by the clinicians, as a way to escape an evaluation which was not acceptable. Other social spaces emerged sometimes as middle grounds. For example, in two cases parents accepted that their children receive therapy at school, but not in a clinical setting. The school was perceived as a less stigmatizing setting. Parents would also research the clinicians’ profiles on the internet and read the team publications and the media coverage of public positions and of the team work. In a few instances, the anti-racist and anti-islamophobia stance of the team helped to strengthen an alliance, but in other cases it reinforced distrust because the team was considered as too “woke”.

#### Perceived consequences of parental extremism

In terms of consequences of the parental/family situation for the child, most clinicians emphasized both the salience of parentification (and pseudo-maturity), and instances of regression, in which the child would act in very disruptive ways to attract adult attention. Risk taking was quite prominent. A clinician felt that a child would consciously provoke him by boasting about adult content he would watch online, identifying through the pride of transgression to his parent. Another reported that a 9-year-old girl was posting sexual pictures on social media, and exhibiting promiscuous behaviors. In some cases contradictory identity issues were central: a mother gave a feminine name to her son, while telling him that he was the son of a deceased male war hero. The clinician reported that this very resilient boy tried to resolve the situation by using a masculine nickname and by giving a central role to a male figure in the sand-play sessions. Some of the children underwent periods of placement in foster families and sometimes separation from parents who fled oversea. Clinicians observed that these placements often introduced even other forms of insecurity and disruption in the children’s lives. Overall sadness, and sometimes death wishes were quite striking to clinicians, who felt that emotional deprivation, parents’ unpredictability and parental alienation were major stressors for many children and that parental separation often aggravated the attachment issues and children’s psychological distress.

Some of the consequences reported by the clinicians were associated with the educational practices of the parents: a six-year-old had to study 2 hours of Arabic before he was allowed to play, a three-year-old boy was prohibited to play and had to watch only propaganda videos, which were very violent. In this last case the non-verbal child was terrified and would hide under the sink, displaying clear startle reactions. Although first involved because of allegations of conjugal violence, youth protection withdrew rapidly after the mother claimed she had lied because she was angry. The family was subsequently lost to follow-up. According to clinicians, many parents adhering to religious extremism (whether converted or not) were quite critical of some school practices (mainly related to food or limited capacity to implement religious practices), but in general they did not critique the teaching. Although the team could never assess their children because they refused, this was not the case of the parents adhering to the alt-right or white supremacist movements who talked at length about the fact that school was brain-washing their children: “Emasculating and castrating their boys, and preparing their daughters to be raped by immigrants”. One of these parents homeschooled all their children, and others wanted to do so but could not because of their spouse’s opposition. All clinicians felt that the school environment was overall very protective for the children because of peer relations, the capacity to escape a toxic or deprived family environment, and because of significant relations with other adults. However, poor relationships between the school and the parents clearly eroded this protection. In a few cases, with the consent of the parents for whom it was more convenient, therapy took place at the school, and this increased its accessibility for the children and decreased the possible stigma and conflict of loyalty.

#### Institutional partnership challenges

Clinicians insisted on the difficulties of bringing together and establishing a consensus among institutional actors around the children: youth protection, the school, the other health care providers, and the security agencies (sometimes). Splitting and conflicts were common, fueled by the strong emotions elicited by representations about radicalization. For example, the team’s clinicians would feel that the parent-child relation was relatively positive and that separation would be harmful, but youth protection professionals and the judge felt that these parents were toxic and dangerous and placed the children in foster care. In other cases, the scenario reverted, with the team expressing much more worry than external professional actors (school, day-care and DYP). Clinicians from the team noted that other professionals tended to have markedly more negative reactions toward religious extremism of parents (these being considered as “terrorists”) than toward other forms of political and gender extremism. In the cases of religious extremism, they tended to minimize the importance of attachment or the protective features of the family, while in the case of majority families adhering to alt-right or white supremacist and masculinist discourses, they would rather minimize the potential risk to the children of being in an environment which demonize the school and legitimizes violence. In such cases, mobilizing child protection to get involved in the situation was particularly difficult, and the clinicians perceived a blind spot around the possible consequences of majority forms of extremism, which were considered by child protection professionals to fall under the normal variation in upbringing practices from which parents can choose, and thus within parental rights.

## Discussion

### Stigmatized and invisible children: the replication of social prejudices in the intervention field?

Although the numbers involved are small, the difference between the frequent reference of children of parents adhering to religious extremism to social and health services by schools and\or security agencies, and the difficulties to reach out to children and youth of other radicalized parents who are members of the majority, is striking. Clinicians confirm the presence of cultural and social stereotypes increasing the representation of minority families as potentially dangerous or inappropriate, and minimizing the perception of risk for parents belonging to the majority. These results confirm the UN Special Rapporteur assertion that “The overregulation and visibility of some families (…) to the security State operates largely along entrenched racial, ethnic and religious lines” (p. 6). These observations may raise concerns for the protection of both minority and majority children. In the case of minority families, profiling and stigma may shatter the family unit and disrupt the child environment, while for majority families, the normalization of certain forms of extremism may expose the child to psychological abuse and to a harmful culture of hate that alienates them from society. After an analysis of a series of radicalization cases appearing in UK family courts, Ahdash (2018; Ahdash, 2020) argues that the interaction between family law and counterterrorism is worrisome as it may become a way to regulate cultural, political, and ideological expressions of Islam. But if the potential harm of this interaction to the protective nature of the family is established and should clearly be avoided, much less is known on the possible harmful consequences of the majority clinicians’ and security agents’ blind spots about the majority forms of extremism on children. As our societies become increasingly polarized, there is an urgent need to learn more about this to strike a balance in the child’s best interest between intervening and stepping back.

### Children in distress: Fragile parents and an unsafe environment

The clinicians reported that the children they assessed or followed were overall experiencing high levels of psychological distress. This psychological distress was mainly related to three sets of issues: family separation, parental psychopathology, and conflicts of loyalty stemming from familial or social alienation.

Arrests, divorce and youth protection decisions provoked repeated painful separations of the children from their caretakers. These, even in the presence of ambivalent attachments, were a source of anxiety and often emotional withdrawal or dissociation in children. This coincides with observations on the children of foreign fighters returning to Europe who were forcibly removed from their parents at arrival and presented high distress and signs of disorganization (Mapelli et al., 2019).

Attachment issues and problems associated with parenting deficits were at the forefront in children of radicalized parents with concurrent psychopathology. This is not surprising as the literature has well established the association between different forms of mental disorder in parents and childhood anxiety, deprivation and sometimes trauma. This was for example reported by Song et al. (2014) in the case of offspring of child soldiers who suffered from their parents’ PTSD. The fact that this sample is a clinical sample and is thus biased and cannot be seen as representing the majority of parents adhering to radicalized ideas should however be kept in mind.

Diagnoses representing the child distress were often ambivalently accepted and negotiated among the children, parents and clinicians. In some cases, parents would look actively for a diagnosis (Attention Deficit Disorder with Hyperactivity or Autism Spectrum Disorder) which would represent the projection of the family problem on the child, in the role of the identified patient. In others, the children and youth would convey that a stress related disorder diagnosis had a soothing effect, by acknowledging the harm they had suffered or were still suffering. Interestingly the clinicians, although they addressed the parent-child relational issues, often refrained from using the Attachment Disorder label (rather favoring the broad stress related disorder category), because of the potential blame it could put on the parents and the need to preserve the therapeutic alliance with them. Overall, diagnoses were not at the forefront of the clinical engagement and were sometimes used by the clinicians as strategic tools to provide a non-threatening explanatory model for the children’s difficulties.

Loyalty conflicts were another major source of distress for the children. This often-involved conflicts between the parents and parental alienation whose harmful impact on children is well established. Conflicts with the school, depicted as toxic and harmful by the parents and ambivalently invested by the child, were also at the forefront. Because the school also appeared to the clinicians to be a relatively protective environment for the children, and a milieu that could monitor, to a certain extent, the child wellbeing, this is of particular importance and suggests that training of school personnel is an urgent need.

The number of children being homeschooled in Canada and in Quebec is increasing steadily (Van Pelt, 2015). Although multiple reasons explain this phenomenon, these numbers may also reflect the fact that an increasing number of extremist anti-system parents in North America are withdrawing their children from schools to home school them, because they disagree with the values and facts being taught at school. The possible consequences of what could be understood as a form of social alienation is becoming a social issue which requires immediate attention.

### Working with children of extremist parents: The clinicians’ challenges

Results illustrate that the establishment of a therapeutic alliance with the parents and the children is fraught with difficulties. Representations about institutional homogeneity and the absence of boundaries between health and security institutions are major stumbling blocks because of the numerous historical and international instances in which clinicians had to surrender confidentiality in the name of national security. Fear of youth protection intrusion in the family and of the clinician’s judgement about parental ideologies are other major sources of distrust. While remaining truthful and refraining from creating an alliance on false premises, the clinicians may need to suspend judgement and sometimes disbelief (Witzum, 1999) in order to establish a relationship around the child and the family wellbeing.

This study has a number of major methodological limitations. First, the small sample does not allow to make any generalization. This clinical sample is clearly biased in terms of parental psychopathology and cannot be seen as representing all situations of children having extremist parents. Furthermore, because of the small sample size, cases could not be presented in depth because of confidentiality issues, and non-aggregated data could not be shared. The fact that the study relies on file data and practitioners’ perceptions does not provide a direct access to children’s and parents’ perspective and entails limited (and some missing) data. In spite of all these limitations, because of the lack of studies in the field, our results provide a possibility to raise important questions and pave the way for further research.

## Conclusion

**Implications for policy**: The results support the need of partnerships emphasizing a multi-actor perspective (education, youth protection, youth mental health, community organizations, security agencies, justice) to address the children of extremist parents’ predicament. Future concertation in the field needs to question jurisprudence in the domain and explore the possible risks and benefits of diverse interactions between family law and national security issues. Examining the possible consequences of social alienation while ensuring a child rights –based approach, and considering some of the potential risks of home-schooling in extremist families (Pašagić, 2019), are some of the major challenges which require immediate attention.

**Implications for practice:** It is urgent to train youth protection and youth mental health practitioners to be aware of their own personal and institutional bias, understand the children predicament and formulate a systemic, trauma and attachment informed intervention plan. Furthermore, training of school is warranted to preserve the protective capacities of school. This would for example entail the promotion of a warm welcoming of the children and an attention to their individual needs, while minimizing conflicts of loyalty for the children by establishing a non-judgemental working alliance with the parents. When worried about a child family because of possible extremist tendencies in the parents, it may be advisable for school professionals to first consult mental health practitioners and youth protection rather than security agencies to minimize stereotyping and loss of community trust and preserve the best interest of the child. Finally, with community organizations, it may be pertinent to explore socialization opportunities across contexts (around school but also involving extended family and community) to prevent children isolation and provide them with different normative systems.

**Implications for research**: trans-sectional and longitudinal studies on the medium- and long-term evolution of children having extremist parents are needed urgently to inform intervention and policies. These should focus on and document the new social and political majority forms of extremism which flourish presently in our polarized societies.
